# *Lentilactobacillus hilgardii* Inoculum, Dry Matter Contents at Harvest and Length of Conservation Affect Fermentation Characteristics and Aerobic Stability of Corn Silage

**DOI:** 10.3389/fmicb.2021.675563

**Published:** 2021-06-02

**Authors:** Francesco Ferrero, Ernesto Tabacco, Giorgio Borreani

**Affiliations:** Department of Agricultural, Forestry and Food Sciences, University of Turin, Turin, Italy

**Keywords:** dry matter, inocula, yeast count, conservation time, aerobic stability

## Abstract

Heterofermentative *Lentilactobacillus hilgardii* isolated from sugarcane silage, has recently been proposed as a silage inoculant to increase aerobic stability. Various conditions can influence the activity of LAB and their ability to alter silage quality (e.g., DM content and length of conservation). The aim of this study has been to evaluate the effect of *L. hilgardii* on the fermentation quality and aerobic stability of whole crop corn silage with different DM contents (from 26 to 45%), conserved for various conservation lengths (13–272 days). The silages were analyzed for their DM content, pH, fermentative profile, microbial count, and aerobic stability. *L. hilgardii* showed a positive effect on improving the aerobic stability of silages, due its ability to produce acetic acid, and reduced the yeast count. The acetic acid content increased as the conservation period increased and decreased as the DM content increased. The yeast count was reduced during conservation in a DM dependent manner and the inoculation with LH determined a reduction in the count of 0.48 log cfu/g. The aerobic stability increased as the conservation period increased, and the treatment with LH on average increased the aerobic stability by 19 h. The results of this experiment suggest that higher aerobic stability could be achieved in corn silages by ensiling at medium or low DM contents, or by increasing the length of conservation if a higher DM content at ensiling is needed. The inoculation with LH helps to improve the aerobic stability of corn silages by reducing the yeast count.

## Introduction

The presence of spoiled silages on a farm is a challenge, and it results in a reduction of the nutritive value of the silage and an increase in the risks to animals and humans (Driehuis et al., 2018). During the feed-out phase, the contact of the mass with air stimulates the growth of the aerobic microorganisms that are most often responsible for the onset of aerobic instability (Borreani et al., 2018). Aerobic deterioration is common in many silages that are opened and exposed to air, with the rate of spoilage being highly dependent on the numbers and activity of the spoilage organisms in the silage and on the fermentative profile of the silo at opening (Muck et al., 2018). It is generall accepted that yeasts play the major role in initiating aerobic spoilage (Pahlow et al., 2003) and the aerobic stability of corn silage has been found to be closely correlated with the number of yeasts (Borreani et al., 2018). Therefore, a fermentative profile with antifungal compounds may be beneficial to avoid aerobic deterioration. Acetic acid has been reported to be an effective compound that improves aerobic stability by reducing yeasts during conservation and by limiting their growth after silage is exposed to air (Kleinschmidt and Kung, 2006; Comino et al., 2014; Ferrero et al., 2019). In the last 20 years, heterofermentative lactic acid bacteria (LAB), and *Lentilactobacillus buchneri* (previously classified as *Lactobacillus buchneri*, Zheng et al., 2020) in particular, have been used as inocula to prevent the aerobic deterioration of silages, because of their ability to produce acetic acid via the anaerobic conversion of moderate amounts of lactic acid to acetic acid and 1,2-propanediol (Oude Elferink et al., 2001; Muck et al., 2018). However, different conditions can influence the activity of LAB and their ability to alter silage quality (Muck et al., 2018), and heterofermentative LAB inoculants have not been consistently effective in improving aerobic stability as a result of the type of forage, the dry matter (DM) of the forage at harvest, variations in the application rate, and the temperature and length of conservation (Kleinschmidt and Kung, 2006; Blajman et al., 2018; Ferrero et al., 2021). Silages with a high DM content (e.g., >38%) are more prone to aerobic spoilage than those with a low content, because the LAB activity is limited by the reduced water activity (a_w_) and, consequently, the production of acids is low (Comino et al., 2014). The length of conservation represents a critical point for the quality and stability of silage. A long conservation period in complete anaerobiosis determines a decrease in the microbial count and in particular in the yeast and mold counts, and consequently improves the aerobic stability of silages, regardless the use of silage inocula (Weinberg and Chen, 2013; Ferrero et al., 2019). *L. buchneri*-based inocula often need a long conservation period (>45 to 60 days) to be efficacious (Driehuis et al., 1999). Producers often want to start feeding-out silages from silos after ensiling periods of less than 30 days, thus making it necessary for the acetic acid to be produced earlier by heterofermentative LAB, during ensiling (Ferrero et al., 2019; Nair et al., 2020). *Lentilactobacillus hilgardii* (previously classified as *Lactobacillus hilgardii*, Zheng et al., 2020), was isolated from sugar cane (Ávila et al., 2014) and deposited and patented as silage inocula (European Patent Application EP2826385A1), thereby becoming a possible new inoculum to improve aerobic stability after a short conservation period. Phylogenetic evaluation and fermentation profiling of *L. hilgardii* have revealed that it is closely related to *L. buchneri*, as both possess the ability to degrade lactic acid to acetic acid and to 1,2-propanediol in anaerobic conditions (Heinl et al., 2012; Drouin et al., 2019). Assis et al. (2014) gave the first indication of the use of *L. hilgardii* as inoculum for corn silage. Subsequent studies were conducted to evaluate the impact of *L. hilgardii* on ensiling fermentation and on the aerobic stability of corn silages, and they reported higher concentrations of acetic acid, lower yeast populations, and a higher aerobic stability than uninoculated silages (Reis et al., 2018; Ferrero et al., 2019; Costa et al., 2021).

Since the DM content at harvest and the length of conservation can affect the silage quality and the effectiveness of inocula, the aim of this study has been to evaluate the effects of *L. hilgardii*, the DM content at harvest and the length of conservation on the fermentation quality and aerobic stability of whole crop corn silages.

## Materials and Methods

### Crop and Ensiling

Thirteen dairy farms operating in the western Po plain, northern Italy, were involved in the research. Each farm was asked to grow the same corn hybrid (*Zea mays* L. cultivar P1517W, Pioneer Hi-Bred Italia Srl, Gadesco Pieve Delmona, Cremona, Italy) and to manage the crop in almost the same agronomic way. Briefly, seeding date in the first decade of April; theoretical planting density of 75,000 seeds/ha; conventional tillage at 0.25–0.30 m depth; fertilization with 20,000–30,000 kg/ha of liquid slurry (average NPK content of 0.27, 0.24, and 0.31% on fresh basis) applied immediately before plowing, 104 kg/ha of potassium chloride (K, 60%) applied prior to planting, 104 kg/ha of diammonium phosphate (NP, 18 and 46%) applied at planting, and 166 kg/ha of urea (N 46%) top dressed at the 6–8 leaf-stage; pre-emergence weed control with Lumax (mesotrione 37.5 g/l, S-metolachlor 37.5 g/l and terbuthylazine, 12.5 g/l, Syngenta, Basel, Switzerland) at a rate of 3.5 l/ha plus an additional post-emergence application with Titus^§^ Mais Extra (nicosulfuron 300 g/kg, rimsulfuron 150 g/kg, DuPont, Milano, Italy) at a rate of 70 g/ha, if necessary; 3–5 irrigations (flooding method, about 200–300 mm each time). In each farm (except one) the crop was harvested at two different maturity stages of growth to obtain different DM contents at ensiling. Corn was harvested, as chopped whole crop, using a precision forage harvester (Claas Jaguar 950, equipped with an 8-row Orbis head, Claas, Harsewinkel, Germany) to a theoretical cutting length of 12 mm. The DM contents of the forages at harvest ranged from 25 to 45%. The field was divided into blocks, which were subsequently harvested separately to obtain three replicates. The fresh herbage of each plot was divided into two piles (about 50 kg), which were either not treated, as a negative control (CONT), or treated with *Lentilactobacillus hilgardii* CNCM I-4785 (Lallemand SAS, France) at 300,000 cfu/g fresh matter (FM) (LH). The lyophilized microbial inoculant was diluted in sterilized water and applied using a hand sprayer, at a rate of 4 mL/kg of forage, by spraying uniformly onto the forage, which was constantly hand mixed. The same amount of water was added to the CONT treatment. In order to add the targeted amount of LAB, the inoculum was plated on MRS agar (Merck, Whitehouse Station, NY), to which natamycin (0.25 g/L) had been added and incubated as described hereafter. Moreover, an appropriate amount of inoculum was used, on the basis of the measured LAB concentration, to achieve the desired application rate.

The fresh forage was sampled prior to ensiling after the inoculum had been applied. The untreated and treated forages were then ensiled (about 10–14 kg of wet forage) in 20 L plastic silos equipped with a lid that only enabled the release of gas. The forages were packed by hand in order to reach the same weight of the silo at similar DM contents. Therefore, the final packing densities, on a wet basis, ranged from 669 to 529 kg FM/m^3^ depending on DM content. All the laboratory silos were filled within 3 h. The silos were weighed and conserved at ambient temperature in a controlled environment (20 ± 1°C) and were then opened after a conservation period ranging from 13 to 272 days, depending on the experiment. On opening, each silo was weighed again, the first 50 mm layer was discarded, and the remaining silage was mixed thoroughly and sub-sampled to determine the DM content, chemical composition, fermentation profile and the microbial counts. The DM losses due to fermentation were calculated as the difference between the weight of the forage placed in each plastic silo at ensiling and the weight of the silage at the end of conservation, corrected for the DM content of the forage and its respective silage.

After sampling, the silages were subjected to an aerobic stability test, which involved monitoring the temperature increases due to the microbial activity in the samples exposed to air in insulated boxes under a controlled environment. About 3 kg from each silo was allowed to aerobically deteriorate at a controlled temperature (20 ± 1°C) in 17 L polystyrene boxes (290 mm diameter and 260 mm height). A single layer of aluminum foil was placed over each box to prevent drying and dust contamination, but also to allow the air to penetrate. The room and silage temperatures were measured hourly by means of a mini temperature logger (Escort Intelligent Mini, Escort Data Logging Systems Ltd., Auckland, New Zealand). Aerobic stability was defined as the number of hours the silage remained stable before its temperature increased by 2°C above the ambient temperature.

### Sample Preparation and Analyses

The pre-ensiled material and the silages were split into five subsamples. One sub-sample was analyzed immediately, for the DM content, by oven drying at 80°C for 24 h. Dry matter was corrected according to Porter and Murray (2001), to consider the volatile compound losses that can take place at 80°C. The second subsample was oven-dried at 65°C to a constant weight and was air equilibrated, weighed and ground in a mill (Cyclotec Tecator, Herndon, VA, United States) to pass a 1 mm screen. The dried samples were analyzed for the total nitrogen (TN), according to the Dumas method (method number 992.23, AOAC International, 2005), using a Primacs SN nitrogen analyzer (Skalar, Breda, The Netherlands), for crude protein (CP) (total N × 6.25) and for ash by ignition (method number 942.05, AOAC International, 2005). The starch concentration was determined according to the AOAC methods (method number 996.11; AOAC International, 2005). Neutral detergent fiber (NDF) was analyzed, using a Raw Fiber Extractor (FIWE, VELP Scientifica, Usmate Velate, Italy), with the addition of heat-stable amylase (A3306, Sigma Chemical Co., St. Louis, MO) and expressed on a DM basis, including residual ash, as described by Van Soest et al. (1991). Acid detergent fiber (ADF) was analyzed and expressed on a DM basis, including residual ash (Robertson and Van Soest, 1981). A third fresh sub-sample was used to determinate the water activity (a_w_), pH, nitrate (NO_3_) and the buffering capacity. a_w_ was measured at 25°C, on a fresh sample, using an AquaLab Series 3TE (Decagon Devices Inc., Pullman, WA), which adopts the chilled mirror dew point technique. The fresh forage was extracted for pH and nitrate using a Stomacher blender (Seward Ltd., Worthing, United Kingdom), for 4 min in distilled water at a 9:1 water-to-sample material (fresh weight) ratio. The total nitrate concentration was determined in the water extract, through semi-quantitative analysis, using Merckoquant test strips (Merck, Darmstadt, Germany; detection limit 100 mg NO_3_/kg DM). The pH was determined using a specific electrode (DL21 Titrator, Mettler Toledo, Milan, IT). The buffering capacity was determined in the water extract, as described by Playne and McDonald (1966). A fourth sub-sample was extracted, using a Stomacher blender, for 4 min in H_2_SO_4_ 0.05 mol/L at a 4:1 acid-to-sample material (fresh weight) ratio. An aliquot of 40 mL of silage acid extract was filtered with a 0.20-μm syringe filter and used for quantification of the fermentation products. The lactic and monocarboxylic acids (acetic, propionic and butyric acids) were determined, by means of high-performance liquid chromatography (HPLC), in the acid extract (Canale et al., 1984). Ethanol and 1,2-propanediol were determined, by means of HPLC, coupled to a refractive index detector, on a Aminex HPX-87H column (Bio-Rad Laboratories, Richmond, CA). The fifth subsample was used for the microbial analyses.

In order to conduct the microbial counts, an aliquot of 30 g was transferred into a sterile homogenization bag, suspended 1:9 w/v in a peptone salt solution (1 g of bacteriological peptone and 9 g of sodium chloride per liter) and homogenized for 4 min in a laboratory Stomacher blender (Seward Ltd., London, United Kingdom). Serial dilutions were prepared, and the yeast and mold numbers were determined using the pour plate technique by inoculating, in duplicate, 1 mL of dilution on Yeast Extract Glucose Chloramphenicol Agar (YGC agar, DIFCO, West Molesey, Surrey, United Kingdom), after incubation at 25°C for 3 and 5 days for yeast and mold, respectively. The yeast and mold colony forming units (cfu) were enumerated separately, according to their macromorphological features, on plates that yielded 15–150 cfu. Since lot of samples had very low yeast and mold counts, when the plates of the original dilution yielded fewer than 15 colonies, actual plate count was recorded and reported as log transformed value. A detection limit of 1.00 log cfu/g (10 cfu/g of silage) has been assumed when plates from original dilution yielded no colony forming unit. The LAB counts were determined on MRS agar, to which natamycin (0.25 g/L) had been added, by incubating the Petri plates at 30°C for 3 days in anaerobic jars with a gas generating system [AnaeroGenTM, Thermo Fisher Scientific, Rodano (MI), Italy]. Since LAB are facultative anaerobe bacteria, anaerobic incubation was chosen to improve the selectivity of the media against *Bacillus* spp. (Spoelstra et al., 1988). The LAB colony forming units were enumerated on plates that yielded 30–300 cfu.

### Statistical Analysis

Data (*n* = 150) pertaining to the inoculum application (CONT, *n* = 75; LH, *n* = 75), the DM content and time of conservation were compared. The data were divided into three classes of DM content, that is, low DM (<33% DM, *n* = 43), medium DM (33–38% DM, *n* = 58) and high DM (>38% DM, *n* = 49). Moreover, the data were divided into four classes of conservation times, that is, very short (<15 days, *n* = 36), short (15–30 days, *n* = 42), medium (31–120 days, *n* = 42) and long (>120 days, *n* = 30). The nitrate content was corrected for the dilution factor and expressed on a DM basis. The microbial counts were log_10_ transformed and presented on a wet weight basis. The values below the detection limit for yeast and mold (detection level: 10 cfu/g of silage) were assigned a value corresponding to half of the detection limit to calculate the average value. An unpaired *t-*test was used to analyze the effect of inoculum application on the fermentative characteristics, microbial counts, chemical characteristics, and aerobic stability. One-way analysis of variance was used to analyze the effect of the conservation time and DM content on the fermentative characteristics, microbial counts, chemical characteristics, and aerobic stability. The data were analyzed using the General Linear Model of the Statistical Package for Social Science (v 26.0, SPSS Inc., Chicago, Illinois, the United States). When the calculated values of F were significant, the Tukey *post hoc* test (*P* < 0.05) was used to interpret any significant differences among the mean values. Multiple regression analysis was used to correlate the acetic acid concentration, yeast count and aerobic stability values with the days of conservation (DAY), silage DM concentration, and treatment (inoculation with LH), using the Draper and Smith (1998) stepwise selection procedure to select the best regression model at a 0.05 probability level. All the determination coefficients (r^2^ or R^2^) reported in this paper were adjusted for degrees of freedom. The data were analyzed across experiments for regression analysis using SPSS v. 26.

## Results

The chemical and microbial characteristics of the corn at ensiling are reported in Table 1. The DM content of the silages ranged from 25.2 to 45.1%. The pH was typical of corn at harvesting, with a mean value of 5.72. a_w_ and the buffering capacity ranged from 0.981 to 0.999 and from 23 to 69 meq/kg DM, respectively. The yeast, mold and LAB counts were on average 6.72, 5.40, and 7.76 log cfu/g, respectively. The nutritional composition of the silages showed a high variability, with starch and NDF ranging from 22 to 37% DM, and from 32 to 49% DM, respectively.

**TABLE 1:** Chemical and microbial characteristics of corn silages at ensiling.

Items	Mean	*SD*	Min	Max
DM (%)	34.7	5.81	25.2	45.1
pH	5.72	0.34	5.54	6.03
NO_3_ (mg/kg DM)	<100	61	<100	156
a_w_	0.992	0.006	0.981	0.999
Buffering capacity (meq/kg DM)	44	15	23	69
Yeast (log cfu/g)	6.72	0.27	6.03	7.37
Mold (log cfu/g)	5.40	0.48	4.54	6.65
Lactic acid bacteria (log cfu/g)	7.76	0.76	5.63	8.74
Crude protein (% DM)	7.1	0.86	5.8	8.3
Ash (% DM)	4.5	0.90	2.8	5.7
Starch (% DM)	30.5	4.20	21.7	37.2
NDF (% DM)	40.2	4.20	32.0	48.8
ADF (% DM)	20.3	2.96	14.9	25.7
Hemicellulose (% DM)	19.9	1.38	17.0	23.2
ADL (% DM)	4.1	0.49	2.9	5.0

**ADF, acid detergent fiber; ADL, lignin; a_w_, activity water; cfu, colony forming unit; DM, dry matter; NDF, neutral detergent fiber; SD, standard deviation.**

The Pearson correlation coefficients of the chemical, fermentative and microbial characteristics of the corn silages are reported in Table 2. The length of conservation was positively correlated with the pH, lactic and acetic acids, 1,2-propanediol, and DM losses. On the other hand, it was negatively correlated with the microbial counts (yeast, mold, and lactic acid bacteria) and fiber component of the silages (NDF, ADF, and hemicellulose). Negative correlations were detected between the DM content and lactic and acetic acids, aerobic stability, and nutritional parameters, except for starch. The DM content was positively correlated with the yeast and mold counts, and starch content. The pH was strongly negatively correlated with the lactic acid content and positively with the LAB count. Acetic acid was negatively correlated with the yeast and mold counts. The DM losses were found to be correlated positively to the days of conservation and to the DM content.

**TABLE 2:** Pearson correlation coefficients of chemical and microbiological characteristics of corn silages (*n* = 150).

	DAY	DM	pH	LA	AA	ET	1,2-PD	Yeast	Mold	LAB	AS	NDF	ADF	HEM	ASH	CP	STARCH
DM	–0.122																
pH	−0.540***	0.143															
LA	0.654***	−0.751***	−0.418***														
AA	0.718***	−0.573***	−0.393***	0.771***													
ET	–0.073	0.220*	0.212*	–0.122	–0.116												
1,2-PD	0.439***	0.021	−0.280**	0.128	0.592***	–0.013											
Yeast	−0.691***	0.551***	0.423***	−0.783***	−0.801***	0.139	−0.410***										
Mold	−0.296**	0.339***	0.186*	−0.392***	−0.388***	0.082	–0.156	0.392***									
LAB	−0.687***	–0.093	0.515***	−0.412***	−0.337***	–0.000	–0.100	0.342***	0.134								
AS	0.600***	−0.345***	−0.254*	0.624***	0.598***	–0.063	0.293**	−0.737***	−0.269**	−0.398***							
NDF	−0.331***	−0.707***	0.101	0.272*	0.218*	–0.050	–0.123	–0.055	−0.185*	0.348***	–0.133						
ADF	−0.214*	−0.764***	–0.018	0.383***	0.291**	–0.020	–0.0915	–0.1565	–0.158	0.262*	0.008	0.953***					
HEM	−0.431***	−0.545***	0.234*	0.100	0.100	–0.082	–0.146	0.076	−0.194*	0.410***	−0.291**	0.927***	0.771***				
ASH	0.127	−0.745***	–0.108	0.569***	0.598***	−0.293**	0.124	−0.473***	−0.368***	–0.019	0.141	0.690***	0.602***	0.709***			
CP	0.288**	−0.575***	−0.266*	0.558***	0.634***	−0.374***	0.231*	−0.477***	−0.369***	−0.188*	0.178*	0.525***	0.455***	0.543***	0.888***		
STARCH	0.091	0.844***	0.167	−0.512***	−0.406***	0.195*	0.060	0.321***	0.219*	–0.154	–0.139	−0.862***	−0.923***	−0.675***	−0.717***	−0.591***	
LOSSES	0.470***	0.403***	−0.347***	0.027	0.171	0.374***	0.400***	–0.153	0.122	−0.401***	0.242*	−0.478***	−0.319***	−0.612***	−0.445***	−0.291**	0.325***

AA, acetic acid; ADF, acid detergent fiber; AS, aerobic stability; DAY, days of conservation; DM, dry matter; ET, ethanol; HEM, hemicellulose; LA, lactic acid; LAB, lactic acid bacteria; NDF, neutral detergent fiber; CP, crude protein; 1,2-PD, 1,2-propanediol.

*P-value < 0.05; **P-value < 0.01; ***P-value < 0.001.

The chemical, fermentative and microbial characteristics of the corn silages, untreated or treated with *L. hilgardii*, are reported in Table 3. The application of inoculum determined higher amounts of acetic acid (*P* = 0.013) and a lower lactic-to-acetic ratio than CONT. 1,2-propanediol was higher in LH than in CONT (*P* < 0.001). The yeast count was lower (*P* = 0.021) and aerobic stability was higher (*P* = 0.044) in LH than in CONT. The DM losses during fermentation and the nutritional parameters were not affected by the application of inoculum. All the other nutritional characteristics of the silages resulted to be unaffected by the LH inoculum.

**TABLE 3:** Chemical, fermentative and microbiological characteristics of corn silages as affected by inoculum.

Items	CONT	LH	SEM	*P*-value
	***n* = 75**	***n* = 75**		
DM (%)	36.4	36.2	0.438	0.718
pH	3.76	3.77	0.006	0.297
Lactic acid (g/kg DM)	49.7	49.6	1.070	0.991
Acetic acid (g/kg DM)	11.9	13.7	0.377	0.013
Lactic-to-acetic ratio	4.3	3.8	0.060	<0.001
Ethanol (g/kg DM)	11.8	12.3	0.313	0.413
1,2-Propanediol (g/kg DM)	0.1	1.3	0.127	<0.001
Yeast (log cfu/g)	3.35	2.83	0.114	0.021
Mold (log cfu/g)	<1.00	<1.00	–	–
LAB (log cfu/g)	7.43	7.88	0.075	0.002
Aerobic stability (h)	96	116	5.005	0.044
DM losses (%)	2.21	2.35	0.047	0.129
Crude protein (% DM)	7.5	7.6	0.082	0.797
Ash (% DM)	4.6	4.8	0.096	0.290
Starch (% DM)	34.0	33.7	0.327	0.729
NDF (% DM)	38.0	38.7	0.361	0.318
ADF (% DM)	19.8	20.2	0.212	0.367
Hemicellulose (% DM)	18.2	18.5	0.171	0.324

**ADF, acid detergent fiber; cfu, colony forming unit; DM, dry matter; LAB, lactic acid bacteria; NDF, neutral detergent fiber; SEM, standard error of the mean*.*

The chemical, fermentative and microbial characteristics of the corn silages for the three classes of DM content are reported in Table 4. The DM content influenced the concentration of fermentative products to a great extent, with a decrease in the lactic and acetic acids from low to high DM silages (*P* < 0.001), even though the pH remained almost constant. On the other hand, the ethanol content increased from low to high DM silages (*P* < 0.001). The yeast count was higher in the high DM silages than in the low DM ones (4.15 vs. 2.17 log cfu/g), and the aerobic stability was higher in the low DM silages than in the medium and high DM ones. The LAB counts decreased slightly as the DM content increased. The high DM silages showed lower (*P* < 0.001) NDF, ADF, hemicellulose and crude protein contents and a higher starch content (*P* < 0.001) than the low DM ones.

**TABLE 4:** Chemical, fermentative and microbiological characteristics of corn silages as affected by DM content.

Items	Low DM <33%	Medium DM 33–38%	High DM >38%	SEM	*P*-value
	***n* = 43**	***n* = 58**	***n* = 49**		
DM (%)	29.4^c^	36.2^b^	42.4^a^	0.438	<0.001
pH	3.75	3.77	3.76	0.006	0.223
Lactic acid (g/kg DM)	61.5^a^	49.1^b^	39.9^c^	1.070	<0.001
Acetic acid (g/kg DM)	15.8^a^	13.6^b^	9.2^c^	0.377	<0.001
Lactic-to-acetic ratio	4.0^b^	3.8^b^	4.5^a^	0.060	<0.001
Ethanol (g/kg DM)	10.9^b^	10.7^b^	14.8^a^	0.313	<0.001
1,2-Propanediol (g/kg DM)	0.57	0.95	0.54	0.127	0.316
Yeast (log cfu/g)	2.17^c^	2.88^b^	4.15^a^	0.114	<0.001
Mold (log cfu/g)	<1.00	<1.00	1.39	–	–
LAB (log cfu/g)	7.94^a^	7.66^ab^	7.39^b^	0.075	0.018
Aerobic stability (h)	141^a^	97^b^	85^b^	5.005	<0.001
DM losses (%)	2.09^b^	2.09^b^	2.68^a^	0.047	<0.001
Crude protein (% DM)	8.0^a^	8.0^a^	6.2^b^	0.082	<0.001
Ash (% DM)	5.3^a^	5.1^a^	2.9^b^	0.096	<0.001
Starch (% DM)	29.9^c^	34.6^b^	37.8^a^	0.327	<0.001
NDF (% DM)	41.6^a^	38.3^b^	33.9^c^	0.361	<0.001
ADF (% DM)	22.4^a^	19.4^b^	17.8^c^	0.212	<0.001
Hemicellulose (% DM)	19.3^a^	18.9^a^	16.1^b^	0.171	<0.001

*Means within a row with different superscripts differ (P < 0.05).*

*ADF, acid detergent fiber; cfu, colony forming unit; DM, dry matter; LAB, lactic acid bacteria; NDF, neutral detergent fiber; SEM, standard error of the mean.*

The chemical, fermentative and microbial characteristics of the corn silages for the four classes of days of conservation are reported in Table 5. The pH decreased as the length of conservation increased (*P* < 0.001). The lactic and acetic acid contents were higher for the Long conservation period than for the shorter period, whereas the lactic-to-acetic ratio decreased as the conservation period increased. 1,2-propanediol was higher than 1.0 g/kg DM after 30 days of conservation. The yeast and LAB counts decreased as the conservation period increased, and the aerobic stability increased from 62 to 159 h. The DM losses increased as the conservation period increased, and indirectly influenced the increase in the crude protein, whereas hemicelluloses decreased and determined a reduction in the NDF content.

**TABLE 5:** Chemical, fermentative and microbiological characteristics of corn silages as affected by time of conservation.

Items	Very short <15 days	Short 15–30 days	Medium 31–120 days	Long >120 days	SEM	*P*-value
	***n* = 36**	***n* = 42**	***n* = 42**	***n* = 30**		
DM (%)	35.6	37.2	36.6	35.3	0.438	0.419
pH	3.82^a^	3.78^b^	3.73^c^	3.71^c^	0.006	<0.001
Lactic acid (g/kg DM)	41.2^c^	44.5^c^	51.8^b^	63.9^a^	1.070	<0.001
Acetic acid (g/kg DM)	9.4^c^	10.7^c^	13.9^b^	18.0^a^	0.377	<0.001
Lactic-to-acetic ratio	4.5^a^	4.3^a^	3.8^b^	3.7^b^	0.060	<0.001
Ethanol (g/kg DM)	11.0^b^	11.8^ab^	12.1^ab^	13.8^a^	0.313	0.030
1,2-Propanediol (g/kg DM)	0.11^c^	0.23^bc^	1.04^ab^	1.73^a^	0.127	<0.001
Yeast (log cfu/g)	4.08^a^	3.44^ab^	2.90^b^	1.68^c^	0.114	<0.001
Mold (log cfu/g)	1.45	1.09	<1.00	<1.00	–	–
LAB (log cfu/g)	8.41^a^	8.19^a^	7.16^b^	6.72^c^	0.075	<0.001
Aerobic stability (h)	62^a^	87^a^	125^b^	159^c^	5.005	<0.001
DM losses (%)	1.95^c^	2.05^c^	2.41^b^	2.84^a^	0.047	<0.001
Crude protein (% DM)	7.2^b^	7.5^b^	7.6^ab^	8.1^a^	0.082	0.025
Ash (% DM)	4.5	4.6	4.7	5.0	0.096	0.389
Starch (% DM)	33.9	33.5	33.9	34.1	0.327	0.906
NDF (% DM)	39.9^a^	38.6^ab^	38.2^ab^	36.2^b^	0.361	0.006
ADF (% DM)	20.7	19.9	19.9	19.3	0.212	0.176
Hemicellulose (% DM)	19.3^a^	18.7^a^	18.2^a^	16.9^b^	0.171	<0.001

*Means within a row with different superscripts differ (P < 0.05).*

*ADF, acid detergent fiber; cfu, colony forming unit; DM, dry matter; LAB, lactic acid bacteria; NDF, neutral detergent fiber; SEM, standard error of the mean.*

The acetic acid, yeast count and aerobic stability regression models, are reported in Table 6 with the days of conservation (DAY), DM content (linear or quadratic, DM or DM^2^) and treatment with *L. hilgardii* (0 = untreated; 1 = treated with LH) as the independent variables. The acetic acid content and yeast count of the silages were influenced by the days of conservation, DM^2^ and treatment, with adjusted coefficients of determination of 0.798 and 0.691, respectively. The application of the LH inoculum determined an increase in the acetic acid (1.73 g/kg DM) and a reduction of the yeast count of 0.48 log cfu/g. The days of conservation influenced the aerobic stability (about 3.7 h for each further 10 days of conservation) and the treatment with LH on average increased for 18.9 h of aerobic stability. On the other hand, the DM content of the silage determined a reduction of 39.6 h every 10% units of increase of DM.

**TABLE 6:** Regression models of acetic acid, yeast count, and aerobic stability on days of conservation, dry matter content and treatment with *L. hilgardii* as independent variables.

	Model	RMSE	Adjusted *R*^2^	*P*-value
Acetic acid (g/kg DM)	y = 16.81 + 0.06012 × DAY – 0.0071 × DM^2^ + 1.731 × TREAT	2.074	0.798	<0.001
Yeast (log cfu/g)	y = 1.478 – 0.0084 × DAY + 0.0023 × DM^2^ – 0.4812 × TREAT	0.775	0.691	<0.001
Aerobic stability (h)	y = 202.3 + 0.3658 × DAY – 3.956 × DM + 18.85 × TREAT	44.9	0.462	<0.001

**DAY, days of conservation; DM, dry matter content (%); TREAT, treatment with L. hilgardii (0 = not treated; 1 = treated); RMSE, root mean square error.**

**FIGURE 1:**
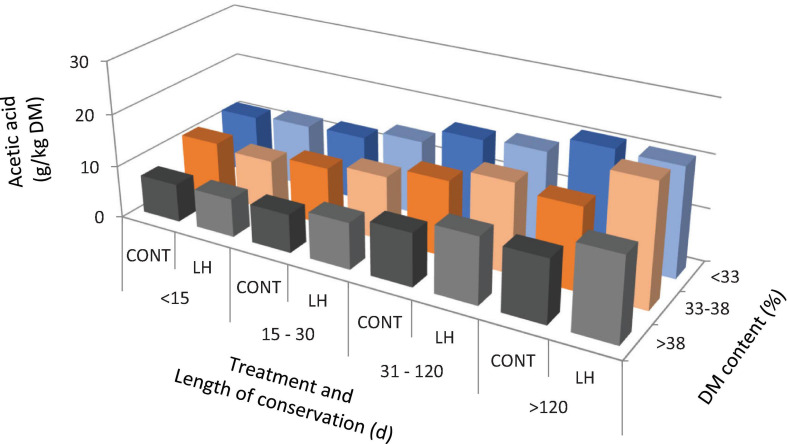
The acetic acid content as affected by treatment with *Lentilactobacillus hilgardii*, DM content and days of conservation.

**FIGURE 2:**
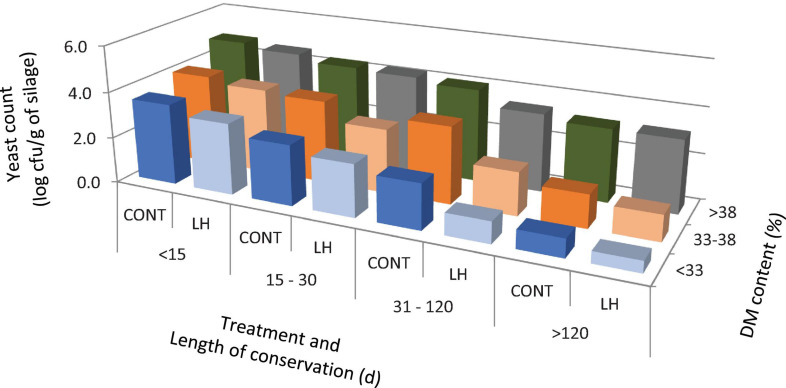
The yeast count as affected by treatment with *Lentilactobacillus hilgardii*, DM content and days of conservation.

**FIGURE 3:**
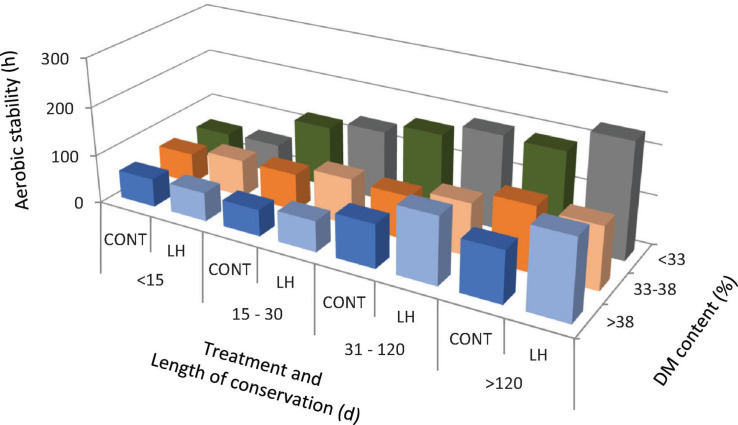
The aerobic stability as affected by treatment with *Lentilactobacillus hilgardii*, DM content and days of conservation.

The acetic acid content, yeast count and aerobic stability, as affected by the treatment with *L. hilgardii*, the DM content and the days of conservation are shown in Figures 1–3, respectively. Acetic acid was influenced by the LH treatment and shows an interaction with the DM and DAY of conservation. The yeast count decreased during conservation, with lower values in low DM silages than in high DM silages, while the silages inoculated with LH presented lower values than C (*P* < 0.001). Aerobic stability was higher in the treated silages than in the untreated ones, higher in the low DM silages than the high DM ones, and higher in the long conservation periods than the short ones (*P* < 0.001).

## Discussion

The present study indicated that the aerobic stability of silage is closely related to the yeast count and, albeit indirectly, to the antifungal activity of acetic acid, and that several interacting factors can influence its magnitude. Heterofermentative LAB inocula have been developed to reduce the yeast count over conservation and to slow down or to inhibit their activity after silo opening, thus improving the aerobic stability of silages (Muck et al., 2018). LAB strains selection is based on their ability to fast growth and to compete with epiphytic microorganisms especially under sub-optimal conditions (e.g., high DM content or low temperature). However, a notable change in LAB composition have been detected during the first fermentation phase (Drouin et al., 2019), and many of the inocula strains are not fast in dominating the fermentation. Under farm conditions, corn is often harvested when the plants are lower or higher in DM than the optimal concentration (from 30 to 38% DM) due to an inadequate capacity to harvest large amounts of forage over a short period of time (Windle et al., 2014). Furthermore, several farmers choose to harvest corn at high concentrations of DM to obtain higher yields and starch concentrations. However, this leads to greater packing difficulties and the resulting silage often spoils rapidly when exposed to air (Comino et al., 2014; Windle et al., 2014). A high DM content, because of a reduced a_w_, limits the LAB activity, with a consequent reduction in volatile fatty acid production (Comino et al., 2014), and in particular of acetic acid, which is known to strongly contrast yeast activity in the presence of air. In our experiment, a restriction of fermentation was observed from wetter to drier silages, with the latter having lower acetic acid concentrations than 10 g/kg DM. This finding is in agreement with the results of Der Bedrosian et al. (2012), who reported that the concentration of acetic acid was higher (14.2 g/kg DM) in 32% DM than in 41% DM (11.6 g/kg DM) corn silage, and of those of Comino et al. (2014), who found a progressive restriction of acetic acid production in corn silage of more than 20 g/kg DM when the DM content increased from 26 to 43%. Lower concentrations of acetic acid in high DM corn silages are considered to be one of the factors that contribute the most to explaining why these silages tend to be less aerobically stable than low DM corn silages (Der Bedrosian et al., 2012). In the present experiment, the lower amount of acetic acid in silages with a higher DM content than 38% resulted in a higher yeast count than 4 log cfu/g. Therefore, in these silages, the aerobic stability was lower than the aerobic stability observed for wetter silages.

Aerobic stability can also be influenced to a great extent by the conservation time. A long conservation period in complete anaerobiosis has been reported to determine a decrease in the yeast count (Weinberg and Chen, 2013; Ferrero et al., 2019). Weinberg and Chen (2013) analyzed the fermentation profile and microbial count of corn silages opened after 1 week to 12 months of conservation. They found an increase in acetic acid of 8–47 g/kg DM, which resulted in a decrease in the yeast count and an improvement in aerobic stability. The same trend pertaining to an acetic acid increase, yeast count decrease and aerobic stability increase was reported by Kleinschmidt and Kung (2006), who investigated untreated corn ensiled at 37% DM, over an ensiling period of 14–361 days. Furthermore, Der Bedrosian et al. (2012) reported that the acetic acid content increased steadily in untreated corn silage with the length of storage, from 9.8 g/kg DM after 45 days of ensiling to 17.1 g/kg DM after 360 days of ensiling. In agreement with the paper mentioned above (Kleinschmidt and Kung, 2006; Der Bedrosian et al., 2012), a long conservation period in the current experiment resulted in an increase in acetic acid content, a reduction in the yeast count and an improvement in aerobic stability. These effects were more evident in the wetter silages than in the drier ones, thus highlighting a positive synergistic effect between the DM of silage and the length of the conservation period. The wetter silages therefore had a lower yeast count and higher aerobic stability than the drier silages for a given length of conservation. This is in agreement with the results of Ferrero et al. (2021) who observed, on corn silages ensiled at 42% DM from 15 to 100 days, that the reduction in the yeast count and the increase in aerobic stability during conservation was less marked than in another experiments (Ferrero et al., 2019) in which corn silages were ensiled, for the same conservation periods but at lower DM contents (36.3 and 34.0%).

Several authors have reported that the heterofermentative LAB *L. buchneri* (the most frequently used heterofermentative LAB inoculum) does not show a consistent effect on corn silages after shorter periods of conservation than 45–60 days (Driehuis et al., 1999; Kleinschmidt and Kung, 2006; Ferrero et al., 2019) or on silages with a higher DM than 38% (Hu et al., 2009; Comino et al., 2014; Xu et al., 2019). To overcome this drawback, recent research has been directed toward finding heterofermentative *Lactobacillus* strains which are able to work rapidly after silo closure. A strain of *L. hilgardii* was isolated from sugarcane silage in Brazil (Ávila et al., 2014) and was produced and industrialized to be active after a short conservation period. Reis et al. (2018) found an increase in acetic acid content after 19 days of ensiling corn silage, but these authors did not perform an aerobic stability test. The same authors however found an increase in aerobic stability in silages treated with LH after 103 days of conservation (Reis et al., 2018). In the study of Ferrero et al. (2019), the addition of LH alone increased the aerobic stability in one out of two corn silage trials, with higher aerobic stabilities observed after 15 and 30 days of conservation. Drouin et al. (2019) found a higher acetic acid production in silages treated with LH than in uninoculated ones already after the first day of ensiling. Costa et al. (2021) found that silages treated with *L. hilgardii* had higher aerobic stability, and lower numbers of yeasts than untreated ones. Ferrero et al. (2021) did not find any differences after 15 or 30 days of conservation between treated and untreated silages, probably because of the DM content of the silages was higher than 40%. In our experiment, *L. hilgardii* was able to dominate the fermentation and to modify the fermentation profile, with a high production of acetic acid and 1,2-propanediol, which is the typical end-product of *L. buchneri* group bacteria (Oude Elferink et al., 2001; Muck et al., 2018). This fermentative profile resulted in a reduction in the yeast count and an average increase in aerobic stability of 20 h, compared to the untreated silages.

In the present paper, we performed a regression model with the days of conservation, the DM content and inoculation with *L. hilgardii* to correlate the acetic acid content, yeast count and aerobic stability. Acetic acid has been found to increase as the conservation period increases and to decrease as the DM content increases. This can be explained by the fact that heterolactic bacteria act after homolactic ones, and their activity is less marked in high DM silages (Pahlow et al., 2003). The inoculation with LH determined an increase in the acetic acid (+1.73 g/kg DM). As expected, the yeast count was reduced as the time of conservation increased in a DM dependent manner, and the inoculation with LH determined an average reduction of the count of 0.48 log cfu/g. The aerobic stability increased by about 3.7 h for every 10 days of conservation, and on average by 18.9 h for the LH treatment, whereas it decreased for an increasing DM content of the silages, as already discussed above, and as reported by several authors (Comino et al., 2014; Ferrero et al., 2021).

The treatment with *L. hilgardii* did not show any differences in the nutritional composition of the silages. On the other hand, the DM content influenced the composition of the corn silage to a great extent. As expected, the increase in the DM content of silage determined an increase in the starch content and a reduction in the NDF content. This can be useful for animal nutrition as starch represents a source of energy for dairy and beef cattle. However, no changes were observed during conservation, although it has been reported that starch degradability increases during storage in corn silages with a high DM content, and that starch degradability increases during storage (Hoffman et al., 2011; Gerlach et al., 2018).

The results of this experiment suggest that higher aerobic stability could be achieved in corn silages by ensiling at medium or low DM contents, or by increasing the length of conservation if a higher DM content at ensiling is needed. The results also suggest that inoculation with *L. hilgardii* helps to improve the aerobic stability of corn silages by enhancing the production of acetic acid, which determines a greater reduction in the yeast counts than those observed in uninoculated silages. The best results have been found in medium DM content silages (i.e., 33–38% DM) and for microbiological stabilized silages (after a medium to long conservation period in complete anaerobiosis, i.e., >30 days). Further study will be needed to evaluate the effect of DM content, length of conservation and treatment with *L. hilgardii* under farm conditions.

## Data Availability Statement

The raw data supporting the conclusions of this article will be made available by the authors, without undue reservation.

## Author Contributions

FF, ET, and GB conceived and designed the experiments. ET and FF analyzed the data. FF wrote the original draft of the manuscript. ET and GB wrote, reviewed, and edited the maunscript. GB supervised, administered the project, and acquired funding. All authors performed the experiments.

## Conflict of Interest

The authors declare that this study received funding from Danstar Ferment AG. The funder was not involved in the study design, collection, analysis, interpretation of data, the writing of this article or the decision to submit it for publication.
